# Phagocytosis of polymeric nanoparticles aided activation of macrophages to increase atherosclerotic plaques in ApoE^−/−^ mice

**DOI:** 10.1186/s12951-021-00863-y

**Published:** 2021-04-28

**Authors:** Tieying Yin, Yanhong Li, Yuzhen Ren, Atik Rohmana Maftuhatul Fuad, Fangfang Hu, Ruolin Du, Yang Wang, Guixue Wang, Yazhou Wang

**Affiliations:** 1 Key Laboratory for Biorheological Science and Technology of Ministry of Education, State and Local Joint Engineering Laboratory for Vascular Implants, Bioengineering College of Chongqing University, Chongqing, 400044 China; 2 School of Medicine, Chongqing University, Chongqing, 400030 China

**Keywords:** Nanomedicine, Atherosclerosis, Macrophages, Phagocytosis, Foam cell

## Abstract

The unique physiochemical properties of nanomaterials have been widely used in drug delivery systems and diagnostic contrast agents. The safety issues of biomaterials with exceptional biocompatibility and hemo-compatibility have also received extensive attention at the nanoscale, especially in cardiovascular disease. Therefore, we conducted a study of the effects of poly (lactic-co-glycolic acid) nanoparticles (PLGA NPs) on the development of aortic atherosclerotic plaques in ApoE^−/−^ mice. The particle size of PLGA NPs was 92.69 ± 3.1 nm and the zeta potential were − 31.6 ± 2.8 mV, with good blood compatibility. ApoE^−/−^ mice were continuously injected with PLGA NPs intravenously for 4 and 12 weeks. Examination of oil red O stained aortic sinuses confirmed that the accumulation of PLGA NPs caused a significantly higher extension of atherosclerotic plaques and increasing the expression of associated inflammatory factors, such as TNF-α and IL-6. The combined exposure of ox-LDL and PLGA NPs accelerated the conversion of macrophages to foam cells. Our results highlight further understanding the interaction between PLGA NPs and the atherosclerotic plaques, which we should consider in future nanomaterial design and pay more attention to the process of using nano-medicines on cardiovascular diseases.

**Supplementary Information:**

The online version contains supplementary material available at 10.1186/s12951-021-00863-y.

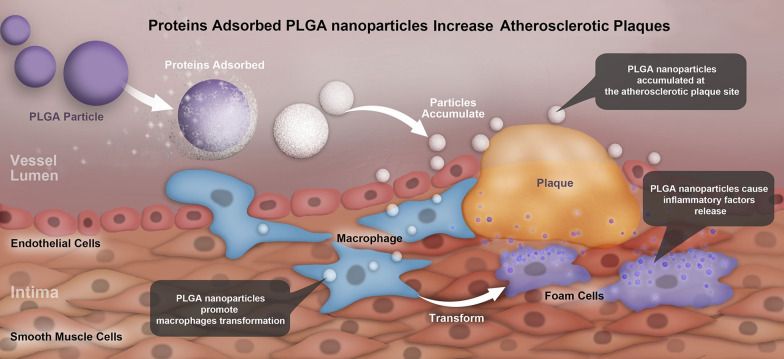

## Research highlights

- Polymeric nanoparticles with exceptional biocompatibility phagocytized by macrophages cause a significantly higher extension of atherosclerotic plaques in ApoE^−/−^ mice.
- Polymeric nanoparticles accumulate at the atherosclerotic plaque and co-localize with the inflammatory site, causing inflammatory factor release.
- Macrophages that are activated and transformed into foam cells, with decreasing cell viability after phagocytizing the accumulated polymeric nanoparticles at the atherosclerotic plaque site.

## Introduction

Nanoparticles (NPs) are ultrafine particles with at least one dimension < 100 nm in size. NPs possess physical properties, such as macroscopic quantum tunneling, nano size and surface effects, which make them desirable for applications in medicine, materials science and biology [1, 2]. NPs may accumulate within the human body through inhalation, ingestion, skin absorption, and injection [3, 4]. The biological safety of nanomaterials has received widespread attention due to their special properties including small size and high specific surface area [5]. An accumulation of NPs in the lungs will result in passage through the alveolar epithelial cells or lymphatic system into the circulation to be redistributed throughout the body. Therefore, nanoparticles may have a significant impact on the cardiovascular system [6–8]. Studies have shown that atmospheric particulate matter, composed mainly of NPs, increases cardiovascular disease morbidity and mortality. The cardiovascular system is now recognized as one of the important targets of nano-toxicity [9, 10].

NPs have more serious biological toxicity and more complex toxicological mechanisms than common chemicals. Studies have shown that NPs can damage vascular endothelial cells (VECs) and trigger an inflammatory reaction, which in turn may cause platelet aggregation and thrombosis [11–13]. Therefore, NPs may be an important risk factor for cardiovascular diseases such as atherosclerosis (AS) [14–16]. Medical research has shown that an inflammatory response is an important pathological mechanism for the development of AS, which can cause endothelial cell dysfunction. When NPs adhere to the cell membrane of VECs, they induce the expression and release of inflammatory factors (such as IL-6, IL-8, and TNF-α) [17, 18]. NPs also may promote adhesion of monocytes to VECs, further differentiation into macrophages, and penetration of the blood vessel walls, leading to AS [19]. Accumulating lipids in unstable plaques further exacerbate the inflammatory response, thereby promoting the development of AS [20]. NPs induce inflammatory reactions, impair lysosomal function, promote abnormal hydrolysis of triglycerides, and lead to an increased lipid load in macrophages, which in turn induces foam cell formation. In the inflammatory state, vascular smooth muscle cells, dendritic cells, and mast cells also may produce foam cells. NPs activate neutrophil elastase, which degrades elastin and various collagens, damaging VECs and basement membranes [21]. NPs interacts with the complement system, coagulation functions and fibrinolysis, which aggravates the formation and instability of arterial plaque [22].

Pober and Cotran examined the relationship between AS and hemodynamics and proposed the shear stress theory to describe the onset of AS [23]. At present, it is well known that atherosclerotic lesions are mainly concentrated in sites with obvious changes in blood flow. In the early stage of plaque development, VECs on the arterial wall attract monocytes, which transform into macrophages and then absorb large amounts of oxidized low-density lipoprotein (ox-LDL) to transform into foam cells. Therefore, atherosclerotic lesions are complex environments containing lipids, cholesterol crystals, inflammatory cells and secreted cytokines. However, when NPs enter the body, they tend to accumulate in areas of infarction. Studies have found that NPs with longer blood circulation times are more likely to cross the endothelial barrier and accumulate in the infarcted area due to the destruction of the endothelial barrier caused by ischemic injury. This mechanism is similar to enhanced permeability and retention [24–26].

In 2007, Dawson and Linse jointly proposed the concept of a protein corona, which led researchers to study the fate of NPs in vivo [27]. NPs will rapidly adsorb proteins forming what is known as the protein “corona” after enters the circulation system. The structure and composition of the protein corona depends on the synthetic identity of the nanomaterial, this would influence the biological identity of NPs [28]. The physiological functions of various proteins that comprise the protein corona generally involve lipid transport, coagulation, complement activation, pathogen recognition, or ion transport [29]. Understanding NPs-protein interactions is a crucial issue in the development of targeted nanomaterial delivery in vivo [30]. The physiological environment to which NPs are first exposed after intravenous administration is the blood stream, and the cell-free portion of the blood (plasma) contains more than 1000 proteins. These proteins potentially interact with NPs to exert different physiological functions, such as recognition by macrophages, causing inflammatory reactions, thrombosis and allergic reactions [31].

The safety issues of biomaterials with exceptional biocompatibility and hemo-compatibility have also received extensive attention at the nanoscale. Numerous nanomaterials have been widely used as drug delivery systems and diagnostic contrast agents in treating cardiovascular diseases. This article focuses on polymer NPs and explores their bioactivity impact on the development of AS and physiochemical mechanisms. Therefore, we conducted a study of the effects of poly (lactic-*co*-glycolic acid) (PLGA) NPs, which are widely used in a variety of Food and Drug Administration approved therapeutic devices, on the development of aortic atherosclerotic plaques in ApoE^−/−^ mice.

## Methods experimental materials and methods

### Experimental materials

PLGA polymer (MW: 90,000, 50:50), 8-week-old C57 BL/6 and ApoE^−/−^ male weighed 20–25 g mice were purchased from Beijing Weitong Lihua Experimental Animal Technology Co.Ltd. (Beijing, China). Male New Zealand white rabbit weighed 3 kg were purchased from Chongqing Daping Hospital Animal Experimental Center (Chongqing, China). The high fat diet contained 0.15% cholesterol and 20% fat. The murine macrophage cell line (Raw 264.7) was purchased from American Type Culture Collection (ATCC, USA).

### Methods

#### Preparation of PLGA and PLGA + PC NPs

PLGA NPs were prepared by a nanoprecipitation proses [32]. Briefly, PLGA (100 mg) was dissolved into 10 mL dimethyl sulfoxide (DMSO). The mixture (2 mL) was precipitated by adding dropwise into 6 mL deionized water with gentle stirring, and further dialyzed using dialysis bag (molecular weight cut-off, MWCO: 3500 Da) against water to remove the free DMSO. The volume was adjusted to 10 mL to obtain PLGA NPs solution (2 mg/mL), collected and preserved at 4 °C. The blood was collected with heparin from the eyeball and stored at 4 °C. The collected blood of the mice was statically placed at 4 ℃ and after 6 h, centrifuged at 3000 rmp for 15 min to obtain serum. To obtain PLGA + protein corona (PC), 1 mL of solution of PLGA (2 mg/mL) was incubated with 1 mL of serum at 37 °C for 30 min.

The samples were collected after three cycles of washing and centrifugation (10 min, 20,000 rpm) in order to remove unbound serum proteins (Additional file 1: Fig. S1). Sodium dodecyl sulfate polyacrylamide gel electrophoresis (SDS-PAGE, Bio-Rad) analysis of the protein corona composition carried out on PLGA and PLGA + PC NPs.

#### Characterization of PLGA and PLGA + PC NPs

The aqueous phase diameter, size and zeta potential of PLGA NPs and PLGA + PC were determined by dynamic light scattering (DLS) using a Malvern Zetasizer Nano ZS unit (Nano ZS 90, Malvern, UK) with He–Ne laser (λ = 633 nm) at a scattering angle of 90° at 25 °C. A drop of PLGA or PLGA + PC NPs solution at a concentration of 100 µg/mL was dropped onto a copper mesh (200 mesh), and air-dried naturally. Then stained by 2 % phosphotungstic acid for 3 min, air-drying. Subsequently, the morphology of PLGA NPs and PLGA + PC were visually observed using a transmission electron microscope (TEM, Zeiss Germany, Optima 75 KV) and scanning electron microscopic (SEM, Thermo Scientific, USA) [33].

#### Determination of serum protein adsorbed by PLGA NPs

PLGA and PLGA + PC NPs were denatured by heating the NPs in SDS sample buffer (Beyotime, China) for 5–10 min at 95 ℃ and separated by electrophoresis on 10% polyacrylamide precast gels. The resulting gels were stained with Coomassie Stain (Bio-Rad) over-night and destained in methanol/water (1:3, v/v) for 12 h. Stained gels were imaged using an ImageQuant LAS4010 image analyzer (GE Healthcare).

Determination of serum protein adsorbed by PLGA NPs was carried out according to the standards bicinchoninic acid (BCA) protein assay kit. PLGA NPs were incubated with 2 mL mouse serum for 30 min, the mixture was centrifuged at 3000 rpm for 20 min, and then the supernatant was collected to determine protein content by the BCA kit. Meantime, untreated serum was the control group.

#### Hemolysis rate of PLGA NPs

The collected fresh vein blood from healthy rabbits were mixed with sodium citrate in a 9:1 ratio to prevent coagulation. Four microliters anticoagulation was added with 5 mL 0.9 % sodium chloride (NaCl) injection to dilute. The first group as a negative control contained 5% glucose, second group as a positive control contained only deionized water and the last group as experimental group consisting of three sub-groups, 2 mg/mL PLGA NPs, 1 mg/mL PLGA NPs and isotonic solution that contained a mixture of PLGA NPs and 5 % glucose.

Then, each group above solution (200 µL) was incubated with 0.9 % NaCl (2.8 mL) at 37 °C for 30 min in a water bath. The mixture was added 60 µL of diluted anticoagulant solution, after second incubation at 37 °C for 60 min in a water bath and centrifugation 3000 rpm for 10 min, the supernatant was collected and measured absorbance (OD) at 545 nm spectrophotometer.

To quantify percent hemolysis, the hemoglobin concentration measured was divided by the hemoglobin concentration of the diluted blood solution as described by the following equation:

1 $$Hemolysis\,rate \left(\%\right)=\left(\frac{OD\,sample-OD\,negative\,control}{OD\,positive\,control-OD\,negative\,control}\right)\times 100 \%$$

Hemolysis rate exceeding 5% is considered hemolysis.

#### Effects of PLGA NPs on APTT, PT, TT and fbg

The blood was collected from healthy rabbits, mixed with sodium citrate in a 9:1 ratio to prevent coagulation. Briefly method, the mixture was centrifuged at 3000 rpm for 10 min, then the top supernatant was collected as platelet-poor-plasma (PPP). The solution (10 µL) was incubated with PPP (300 µL) at 37 °C for 30 min. Finally, the incubated mixture was conducted evaluation of effects of NPs on plasma coagulation include activated partial thromboplastin time (APTT), prothrombin time (PT), thrombin time (TT), and fibrinogen (Fbg) levels using a fully automated coagulation apparatus.

#### Activation of platelet α-granule membrane protein (GMP-140) by PLGA NPs

Rabbit venous blood was anticoagulated with sodium citrate in a ratio of 9:1, centrifuged at 1000 rpm for 10 min, and the supernatant was collected to obtain as platelet-rich plasma (PRP). Then, the PLGA NPs solution (10 µL) was incubated with PRP (300 µL) at 37 °C for 30 min, the incubated mixture was tested with ELISA kit.

#### Animal experiment

Army Medical University Animal Experiment Ethics Committee and Authority approved all animal procedures for Animal Protection. ApoE^−/−^ and C57 mice were used in this study in accordance with the guidelines of the Chinese Animal Care and Use Committee standards.

The experimental animals were fed with an adaptive feeding week. As shown in Table 1, twenty C57 mice were randomized into two groups, and forty ApoE^−/−^ mice were randomized into four groups (10 mice per group). Then, the mice were subjected to the different treatments for 4 and 12 weeks. PLGA NPs was injected at a dose of 10 mg/kg and the frequency of injection was once every 2 days. The control group was injected with 150 µL 5% glucose isotonic solution. During the experiment, all the experimental animals were fed with high-fat diet, freely drinking water [34]. In order to ensure the success rate of injection, we used a special tail vein instrument (Zhenhuabio, China).

After treatment for 4 and 12 weeks the serum, from the mice were harvested. Total cholesterol (TC), triglyceride (TG), high density lipoprotein (HDL-C) and low-density lipoprotein (LDL-C) were detected using an automated biochemical analyzer.

**Table 1:** Group of experimental animals

Strain	Processing method	Name	Injection frequency	Injection dose	Quantity
C57	12w-HFD + Glu	C57	Once every 2 days	150 µL	10
C57	12w-HFD + PLGA	C57 + NPs	Once every 2 days	10 mg/kg	10
ApoE^−/−^	12w-HFD + Glu	ApoE^−/−^(L)	Once every 2 days	150 µL	10
ApoE^−/−^	12w-HFD + PLGA	ApoE^−/−^+NPs(L)	Once every 2 days	10 mg/kg	10
ApoE^−/−^	8w-HFD + 4w-Glu	ApoE^−/−^(S)	Once every 2 days	150 µL	10
ApoE^−/−^	8w-HFD + 4w-PLGA	ApoE^−/−^+NPs(S)	Once every 2 days	10 mg/kg	10

#### Analysis of atherosclerotic plaques

ORO staining of the cross-sections of the aortic roots was performed as previously described [35]. After treatment for 4 and 12 weeks the aortas, from the heart to the iliac bifurcation, from the mice were harvested. Aortas were fixed by perfusion with paraformaldehyde (4% in PBS). After removing the periadventitial tissue, aortas were dissected longitudinally, and then stained with oil red O (ORO) to quantify the plaque area. The extent of atherosclerotic plaque at the aortic root was also determined by ORO staining.

#### Histology and immunohistochemistry staining of the aortic root

Histology and immunofluorescence staining of the cross-sections of the aortic roots was performed as previously described [35]. The aortic roots were fixed with paraformaldehyde (4% in PBS) for 1 h, and then prepared to paraffin sections. After dewaxing, Masson’s trichrome and hematoxylin and eosin (H&E) staining were used to observe the collagen, lipid core and some plaque ruptures. For immunohistochemistry analysis, the activity of the endogenous peroxidase was inhibited by immersion into 3% hydrogen peroxide and 100% methanol for 20 min. Then, the sections were blocked with 5% bovine serum albumin in PBS for 60 min. Antibodies to TNF-α, IL-6, IL-10. Sections of the main organs including heart, liver, spleen, lung, and kidney were also analyzed by H&E staining.

#### PLGA NPs co‐localization with the inflammatory plaque site

DiI@PLGA NPs solution (2 mg/mL) was prepared by the similar preparation method of PLGA NPs described in previous part, mixing DiI solution (1 mM, 15 µL) and PLGA (15 mg) dissolved in 1 mL DMSO. Then the DMSO is removed. The volume was adjusted to 7.5 mL to obtain 2 mg/mL DiI@PLGA NPs solution. C57 mice were control group, and ApoE^−/−^ mice were experimental group (3 mice per group). The experimental animals were fed with HFD for 12 weeks, then DiI@PLGA (200 µL) was injected through the tail vein. After 24 h, mice were euthanized, perfused with PBS containing 4% paraformaldehyde and heparin sodium, and the heart was isolated. Immunofluorescence staining of the cross-sections of the aortic roots was performed as previously described [35]. The frozen sections of carotid roots were incubated with 5% serum. Then, the sections were incubated with anti-CD68 and CD11b antibody overnight at 4 °C, followed by Donkey anti-rabbit IgG H&L for 2 h at room temperature. Samples were stained with DAPI to show the cell nucleus. The sections were observed by the confocal laser scanning microscopy (SP8, Leica, Germany).

#### PLGA NPs co-culture with Raw264.7 cells

Raw 264.7 cells were cultured in DMEM medium containing 10% fetal bovine serum (FBS) at 37 °C with 5 % CO_2_. After 6 h incubation, the first medium was discarded. The cells were starved for 12 h, then treated with different concentrations (0, 50, 100, 200, 400 µg/mL) of PLGA NPs and PLGA + PC, and added DMEM without serum [36]. After incubating in a 37 ℃ 5% CO_2_ incubator for different times (4, 12, 24, and 48 h), 20 µL of MTS assay solution was added to each well, and incubation was continued for 1 h. The absorbance (OD) of each well was measured at a wavelength of 490 nm using a microplate reader, and repeated six times at each time point. Cell viability was obtained by the following equation:

2 $$\text{Cell viability} = (\text{ODT}-\text{ODB})/(\text{ODC}-\text{ODB}) \times 100\%$$

ODT indicates the absorbance of the experimental group; ODC indicates the absorbance of the control group; and ODB indicates the blank absorbance.

#### PLGA NPs phagocytized by Raw264.7 cells

Raw264.7 cells were seeded in 12-well plates at a density of 2 × 10^5^ cells per well in 1 mL of DMEM medium containing 10 % FBS, and cultured at 37 °C with 5 % CO_2_ for 24 h. 100 µg of DiI@PLGA and DiI@PLGA + PC were added. After incubation for different times (0.5, 2 and 4 h), the cells were washed with PBS, and fixed with paraformaldehyde (4 % in PBS). The nuclei of the cells were stained with DAPI. The cells were observed using the confocal laser scanning microscopy (CLSM).

The same experiment of Raw264.7 cells were also carried out in 6-well plates, for the quantitative analysis of PLGA NPs engulfment by flow cytometry.

#### Effect of PLGA NPs on the transformation of macrophages to foam cells

Raw264.7 cells were seeded in 6-well plates at a density of 2 × 10^5^ cells per well in 1 mL of DMEM medium containing 10% FBS, and cultured at 37 °C with 5 % CO_2_ for 24 h. Different concentrations (0, 50, 100, 200, 400, 500 µg/mL) of PLGA NPs and PLGA + PC were used to treat Raw264.7 cells simulated with ox-LDL (50 mg/L). After incubation for 48 h, ORO staining was performed of the treated Raw264.7 cells, then observed and photographed using a microscope. Other treated Raw264.7 cells were collected by trypsinization centrifuged at 1000 rpm for 10 min, ultrasonic crushing 1 min. Total cholesterol (TC) and free cholesterol (FC) were measured according to the TC kit and FC kit methods, and the protein content was determined according to the BCA kit method. The content of various cholesterol in Raw264.7 cells was expressed by TC and FC per gram of cellular protein. Each experiment was repeated 3 times [37].

## Results

### Characterization and blood compatibility of PLGA NPs

**Fig. 1:**
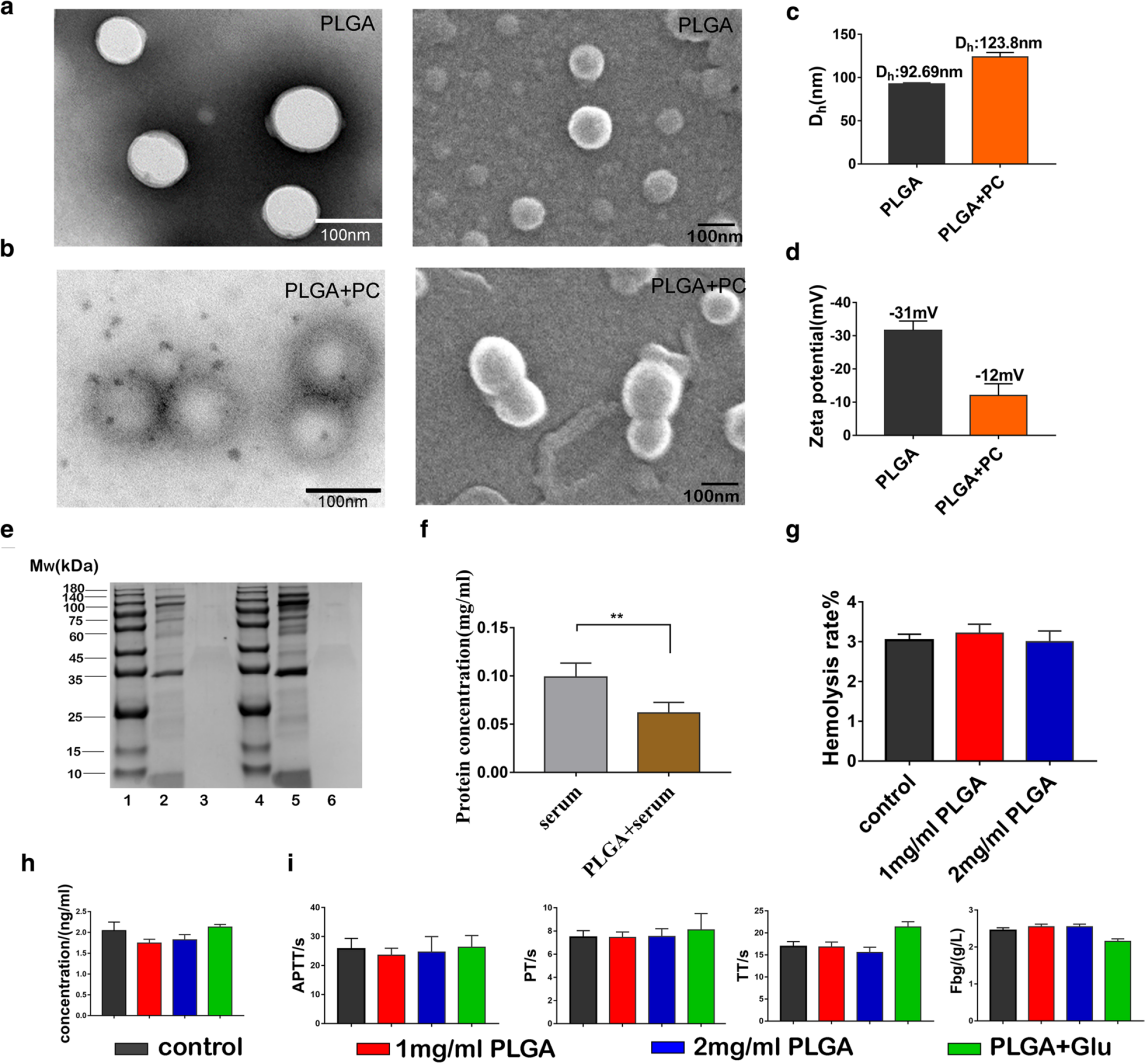
Characterization and blood compatibility of PLGA NPs. TEM and SEM morphology of **a** PLGA NPs and **b** PLGA + PC. **c** DLS particle size and **d** electric potential results for PLGA NPs and PLGA + PC. **e** SDS-PAGE: Characterization of surface bound protein coronae formed on PLGA and PLGA + PC exposed to serum. Lane 1: standard molecular marker, Lane 2: 50 µg PLGA + PC, Lane 3: 50 µg PLGA, Lane 4: standard molecular marker, Lane 5: 100 µg PLGA + PC, Lane 6: 100 µg PLGA. **f** PLGA NPs adsorb proteins in serum to form the protein corona. **g** The hemolysis rate of PLGA NPs. **h** The concentration of α-granule membrane protein in platelets and **i** Response of coagulation variables (APTT, PT, TT, Fbg) after PLGA NPs were added. n = 3, mean ± SD, **P < 0.01

The DLS results showed that the diameters of PLGA and PLGA + PC in the water phase were 92.7 ± 3.1 nm and 123.8 ± 5.3 nm, respectively. The Zeta potentials were − 31.6 ± 2.8 mV and − 12.0 ± 3.5 mV, respectively (Fig. 1c, d). PLGA + PC particles in an aqueous solution were larger than PLGA NPs alone and were less stable. Under dry conditions, TEM and SEM showed that PLGA and PLGA + PC were spherical particles consistent in size with the DLS measurements. Furthermore, we also found that PLGA + PC is more likely to aggregate two or more than PLGA by SEM, which may be related to the formation of the plasma protein corona on its surface (Fig. 1a, b). Serum protein concentrations, measured by BCA kit, decreased after incubation and confirmed the presence of a protein corona on the PLGA NPs due to the formation of PLGA + PC (Fig. 1f). The molecular weight distribution pattern of proteins and protein complexes shown in the SDS-PAGE images revealed that the corona was composed of a large number of different constituents (Fig. 1e).

As shown in Fig. 1g, the hemolysis rates of 1 mg/mL PLGA NPs, 2 mg/mL PLGA NPs, and PLGA + Glu were 2.96 ± 0.10%, 3.24 ± 0.14%, and 2.95 ± 0.29%, respectively. Hemolysis rates in three experimental groups were less than 5%, according to the national standard for the hemolysis rate of medical biological materials. There were no significant differences in GMP-140, APTT, PT, TT and Fbg values between the negative control group and the experimental groups, indicating that PLGA NPs had no significant effect on coagulation and did not induce platelet activation (Fig. 1h, i).

### PLGA NPs cause a significantly higher extension of atherosclerotic lesions in ApoE^−/−^ mice

**Fig. 2:**
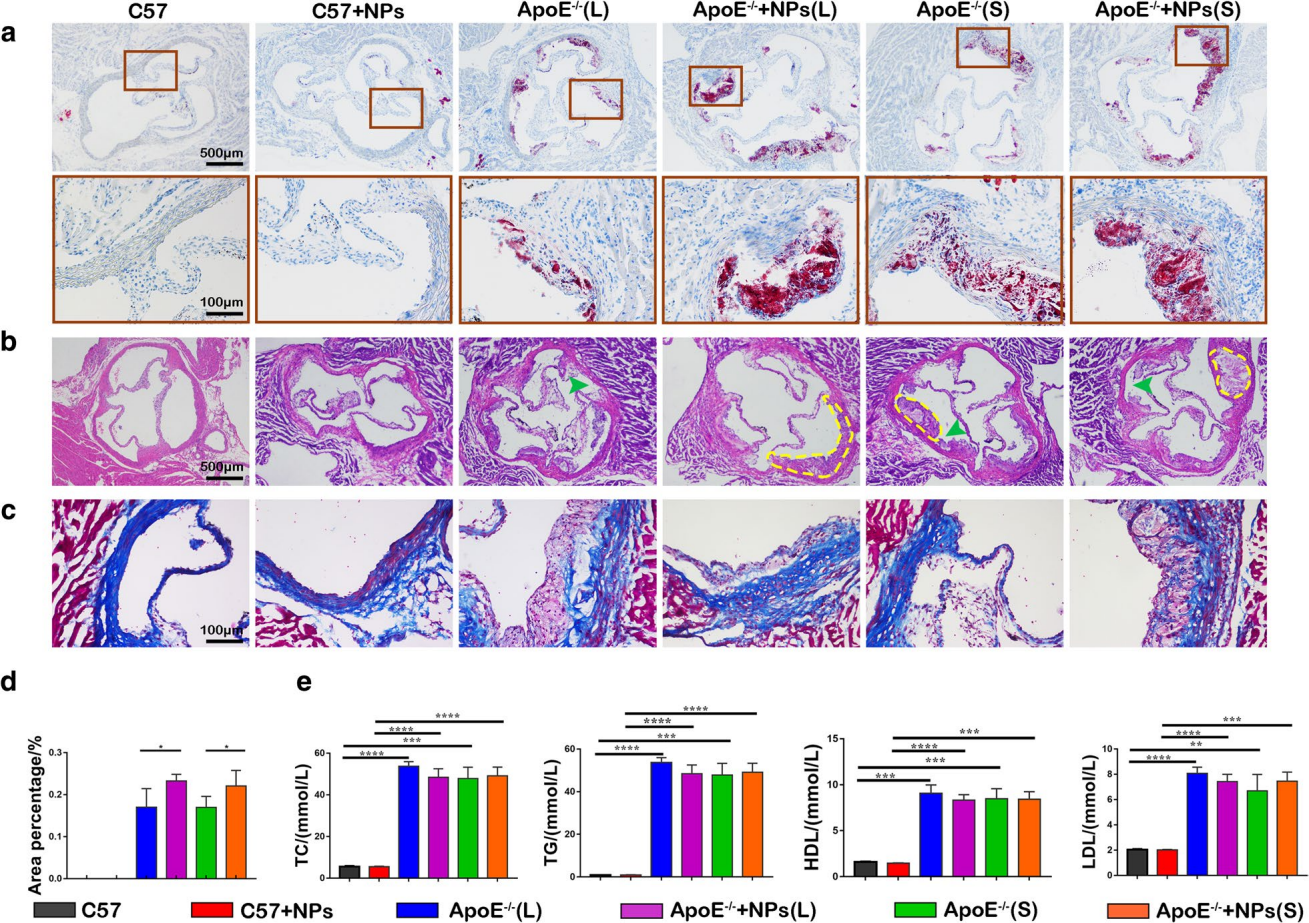
The combination of PLGA NPs and a high-fat diet causes a significantly higher extension of plaques in ApoE^−/−^ mice. **a** ORO stained images of aortic sinus tissues in ApoE^−/−^ and C57 mice. **b** H&E stained images of aortic lipid cores (yellow dashed line and plaque ruptures (green arrows) in ApoE^−/−^ and C57 mice. **c** Images of collagen in the plaque areas stained by Masson’s trichrome. **d** Quantitative data of the atherosclerotic plaque area in the aortic root sections. **e** The lipid assays from serum during atherosclerotic development (TC, TG, LDL, HDL). n = 5, mean ± SD, *P < 0.05; **P < 0.01; ***P < 0.001; ****P < 0.0001

The in vivo effects of PLGA NPs on the development of atherosclerotic lesions were investigated by continuous PLGA NPs injection and administration of a HFD to ApoE^−/−^ mice. Atherosclerotic plaque formation was detected in the aortic sinus, with a large number of red fat granules accumulating in the ApoE^−/−^ mice groups compared to the C57 control groups (Fig. 2 a). The areas of plaque and lipid deposition were severe in ApoE^−/−^ mice with PLGA NPs injection groups, 23.24 ± 0.8% vs. 16.99 ± 1.8%, 22.03 ± 1.4% vs. 16.95 ± 1.1%, 4 and 12 weeks respectively (Fig. 2d) (*P* < 0.05). While there were no changes in all C57 groups. These results suggested that the combination of PLGA NPs and HFD caused a significantly higher extension of plaques after 4 and 12 weeks of continuous administrations in ApoE^−/−^ mice.

Next, we examined the composition of atherosclerotic plaques in aortic root sections by immunohistochemistry staining. H&E staining of aortic sinuses in ApoE^−/−^ mice revealed extensive plaque formation and severe stenosis in the lumen (Fig. 2b). Thickening of the intima and irregular bulging in the lumen, an increase in cell hyperplasia and atherosclerotic plaque formation below the intima were observed. The structure of the arterial wall was disordered, the media was contracted and atrophied, and ruptured plaques with lipid cores were apparent. The collagen arrangement in the aortic sinuses of each group of ApoE^−/−^ mice was disordered, and collagen fibers in the long-term injection of PLGA NPs group were scattered (Fig. 2c).

Hyperlipidemia is known to play an important role in the process of plaque formation. Therefore, we investigated the effect of continuous injection of PLGA NPs on hyperlipidemia. However, with the exception of HDL-C, there were no significant differences in other lipid profiles between the HFD and PLGA NPs groups, only statistically significant differences between ApoE^−/−^ and C57 mice groups. (Fig. 2e). Serum TC in the ApoE^−/−^ mice group was approximately sevenfold higher than in the C57 wild type group; TG was nearly 50-fold higher, and LDL-C and HDL-C were fourfold higher. These observations indicated that PLGA NPs did not cause significant changes in blood serum lipids during the formation of AS in ApoE^−/−^ mice and also the wild type C57 mice. In addition, the H&E staining method was used to observe the pathological sections of the main organs such as myocardium, liver, spleen, lung and kidney after injection of PLGA NPs into the tail vein to determine the possible effects of PLGA NPs on the main organs of experimental animals. There were no obvious changes of the main organs in all experimental animals, which further confirmed their biocompatibility (Additional file 1: Fig. S2).

### PLGA NPs co‐localization within the inflammatory plaque site

**Fig. 3:**
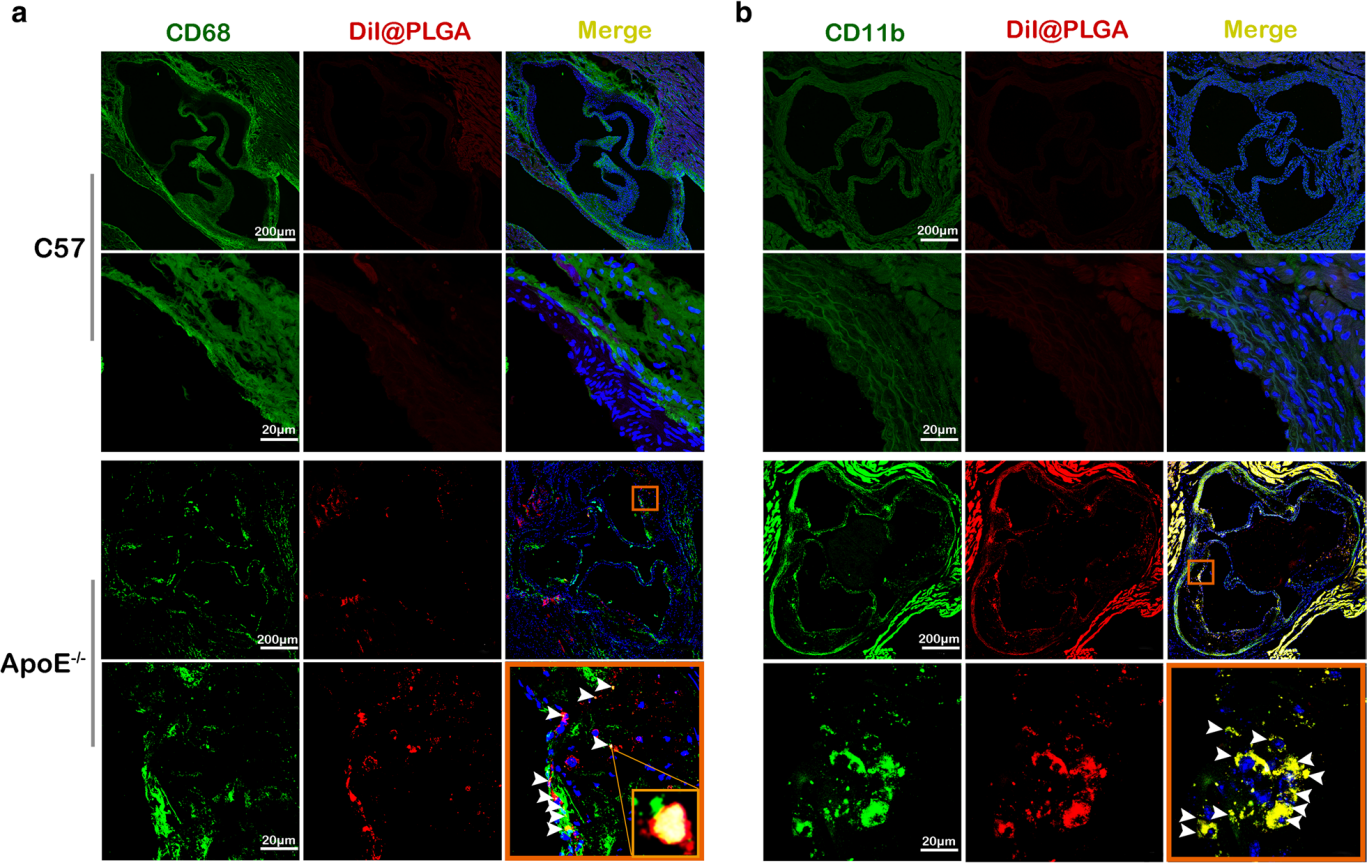
PLGA NPs are enriched at plaque sites (red) and co-localize with inflammatory cells (yellow). The experimental animals were fed with HFD for 12 weeks, then DiI@PLGA (2 mg/mL, 200 µL) was injected through the tail vein and DiI@PLGA were detected in arterial plaque 24 h after injection into the blood stream. CLSM images of the PLGA NPs co-localized with **a** CD68 positive cells and **b** CD11b positive cells (white arrow heads)

Atherosclerosis is characterized by plaque formation and chronic inflammation of the arterial wall. We detected accumulation of NPs at the site of the plaque and co-localization of PLGA NPs at sites of inflammation by immunofluorescence staining. DiI@PLGA NPs were detected in arterial plaque 24 h after injection into the blood stream. CD68 is a marker for a wide range of macrophages that can effectively label monocytes and macrophages (Fig. 3a). CD11b can label neutrophils, monocytes, and macrophages, and function in adhesion and signal transduction during the inflammatory response (Fig. 3b). The results showed that the PLGA NPs co-localized in the plaque sites with CD68 and CD11b positive cells, indicating that the PLGA NPs had a close relationship with AS inflammation, especially the macrophages. We speculate that the NPs co-localized in the plaque sites because they were detected as foreign objects that stimulated macrophages, causing an inflammatory reaction, and increasing phagocytosis inside the plaque.

### PLGA NPs cause inflammatory factor release

**Fig. 4:**
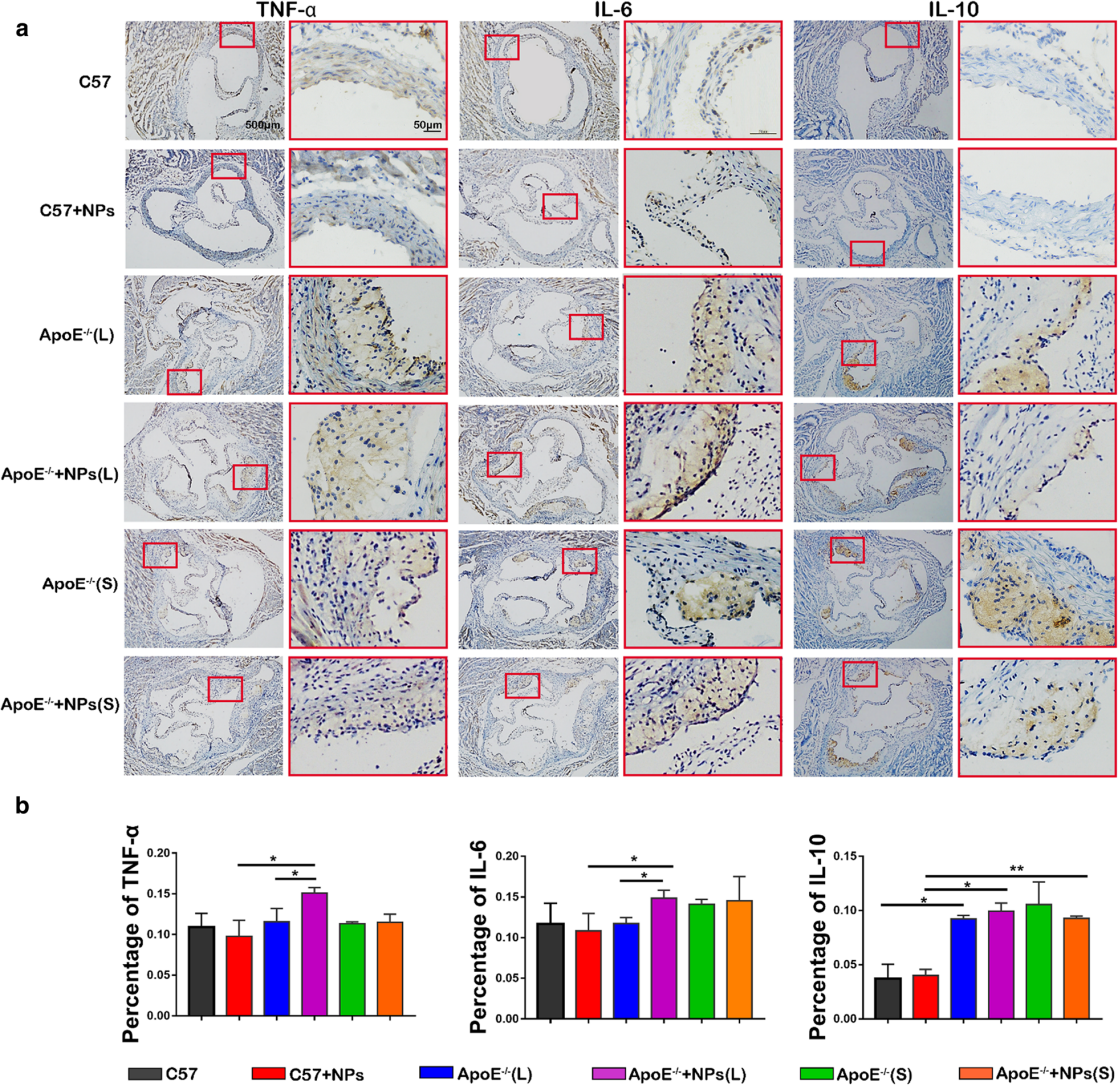
PLGA NPs cause an increasing inflammatory factor release in ApoE^−/−^ mice on a HFD and long-term injection. **a** Representative images of immuno-histochemistry staining with antibodies to TNF-α, IL-6 and IL-10. **b** Quantitative data of TNF-α, IL-6 and IL-10 in plaque areas of the aortic root sections. n = 4, mean ± SD, *P < 0.05; **P < 0.01

After confirming that PLGA NPs co-localized with inflammatory cells in atherosclerotic plaques, we investigated the effects of PLGA NPs injection on the expression of the inflammatory factors TNF-α, IL-6 and IL-10 (Fig. 4a). Positive expression of TNF-α and IL-6 was indicated by brown particles located mainly in the cytoplasm of endothelial and smooth muscle cells. Expression was pronounced in the plaques of the 12-week PLGA NPs injection groups as indicated by dark brown staining. The expression of IL-10 was strongly positive at plaque sites in the ApoE^−/−^ mice groups compared with that in the C57 mice groups, but no significant differences were found between each PLGA NPs injection group and their respective controls (Fig. 4b). These observations indicate that under the influence of a HFD, continuous long-term injection of PLGA NPs can promote an inflammatory response in the plaques of ApoE^−/−^ mice, and PLGA NPs coupled with a HFD had a long-term synergistic effect on the production of AS lesions.

### PLGA NPs were phagocytized by macrophages and decreased cell viability

**Fig. 5:**
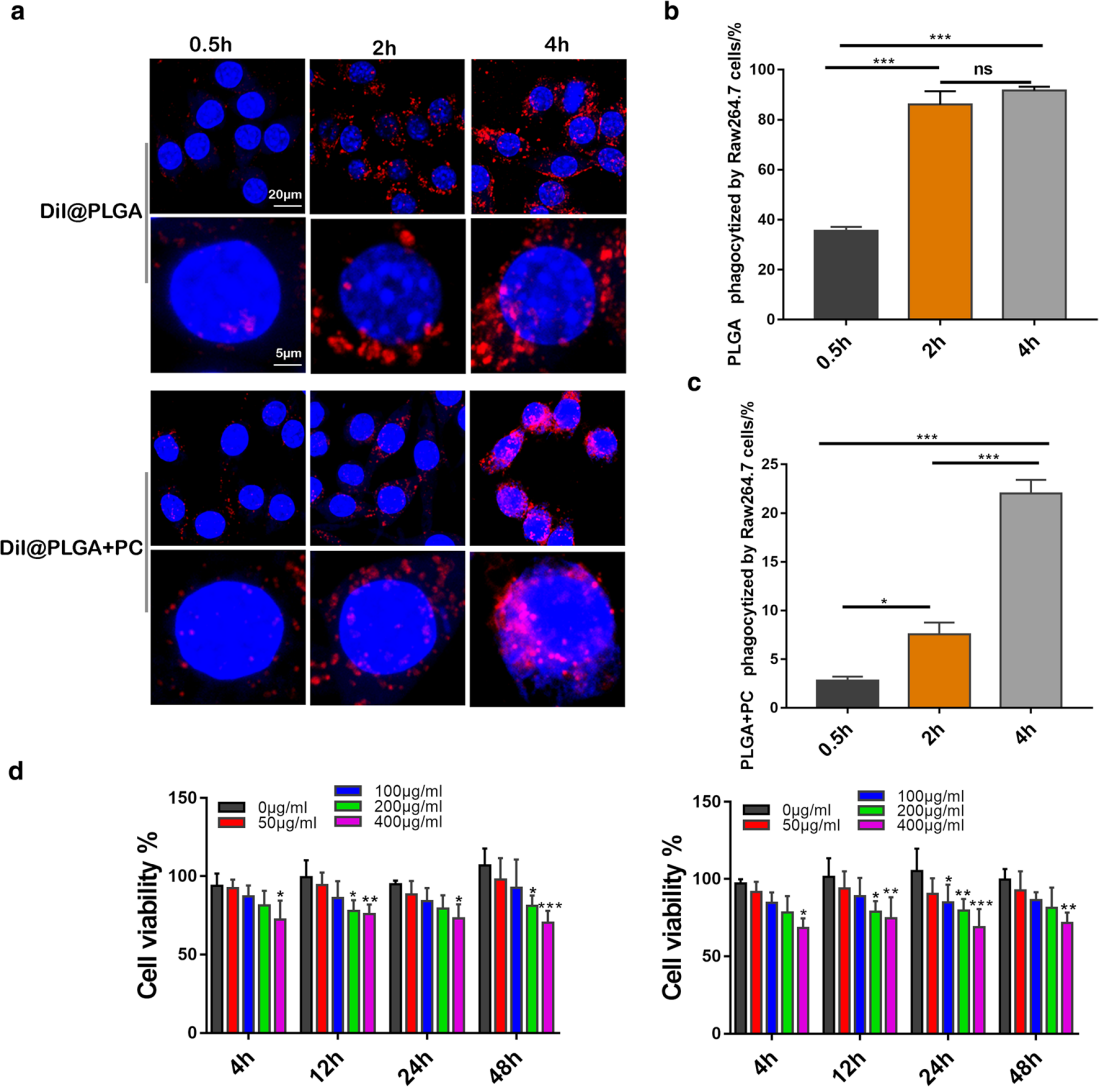
Effects of PLGA NPs and their protein coronas on macrophage activity and phagocytosis. **a** CLSM images of DiI@PLGA and DiI@PLGA + PC phagocytosed by Raw 264.7 macrophages at different time points. Quantification of cellular phagocytosis of **b** DiI@PLGA and **c** DiI@PLGA + PC in Raw 264.7 macrophages at different time points by flow cytometry. **d** Quantitative data of Raw 264.7 macrophage cell viability in different concentrations of PLGA and PLGA + PC NPs. n = 6, mean ± SD, **P < 0.01; ***P < 0.001; *ns* not significant

Macrophages are key contributors to the atherosclerotic process due to their inflammatory and phagocytosis inducing properties. The dynamic phagocytosis of NPs by macrophages was investigated using DiI loaded NPs and observed under confocal laser scanning microscopy (CLSM) and flow cytometry. Raw 264.7 cells began to phagocytize PLGA NPs and PLGA + PC from 0.5 h and increased over time (Fig. 5a–c). The red fluorescence in the PLGA + PC group was more evident in the nucleus compared to the PLGA NPs group. Presumably, the protein corona on the PLGA NPs surface was more likely to help the NPs enter the nucleus (Fig. 5a). The amount of phagocytosis after treatment with NPs for 0.5 h by DiI@PLGA and DiI@PLGA + PC was 35.8 and 2.57%, respectively; after 2 h, phagocytosis increased to 88.2 and 7.72%, respectively; then increased to 92.0 and 22.9% after 4 h, respectively (Fig. 5b, c).

To confirm whether the accumulated PLGA NPs would be phagocytized by macrophages and influence their function, we conducted cell viability assays of Raw 264.7 macrophages co-cultured with different PLGA NPs concentrations. The activity of Raw 264.7 cells decreased but remained greater than 60 % using different concentrations of PLGA NPs and PLGA + PC treated cells (Fig. 5a, b). When the concentration was less than 100 µg/mL, we observed no significant effects on the viability of Raw 264.7 cells. However, when the concentration was greater than 200 µg/mL, the viability of Raw 264.7 cells decreased significantly at all-time points except 4 h. These observations indicated that PLGA NPs and PLGA + PC affect Raw 264.7 cell viability in a time and dose-dependent manner.

### PLGA NPs promote macrophage transformation to foam cells

**Fig. 6:**
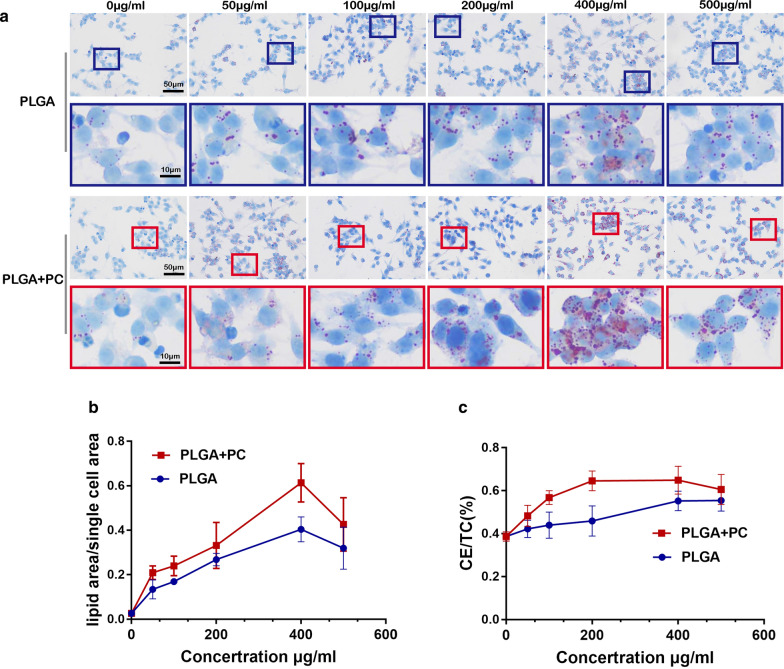
Effects of PLGA NPs and their protein coronas on the transformation of macrophages into foam cells. **a**, **b** Effects of PLGA NPs and PLGA + PC on the phagocytosis of ox-LDL by Raw 264.7 macrophages. **c** The CE/TC (%) of macrophages to foam cells after treatment with NPs. n = 3, mean ± SD

One of the typical pathological hallmarks of AS is the excessive accumulation of ox-LDL and cholesteryl esters in macrophages and their conversion to foam cells. After accumulating in atherosclerotic plagues, PLGA NPs phagocytized by macrophages will influence the formation of foam cells. To investigate the effects of PLGA NPs and PLGA + PC on macrophage transformation to foam cells, we first treated Raw 264.7 cells with 50 µg/mL ox-LDL and then treated the cells with PLGA NPs or PLGA + PC at different concentrations (0, 50, 100, 200, 400, 500 µg/mL) for 48 h. PLGA NPs and PLGA + PC both promoted the phagocytosis of ox-LDL by Raw 264.7 cells, and reached a maximum at 400 µg/mL. The phagocytosis of ox-LDL by Raw 264.7 cells decreased when the nanoparticle concentration was 500 µg/mL compared with 400 µg/mL, probably because the phagocytosis of ox-LDL had reached saturation and NPs could no longer produce an effect. Compared with PLGA NPs, PLGA + PC more strongly promoted the phagocytosis of ox-LDL into Raw 264.7 cells at the same concentration (Fig. 6a, b). NPs also increased the Raw 264.7 CE/TC values (%) at higher concentrations but showed no significant differences at concentrations less than 100 µg/mL (Fig. 6c). These findings confirmed that PLGA NPs and PLGA + PC will accelerate the transformation of Raw 264.7 macrophages into foam cells, and that PLGA + PC has a stronger effect than PLGA NPs.

## Discussion

Due to their unique physico-chemical characteristics, advantages of NPs in this context include their ability to easily penetrate across cell barriers, preferential accumulation in specific organelles and cells, and theranostic (both therapy and diagnostic) properties, as well as their capacity for fine tuning. Polymer NPs are attracting attention due to high efficiency, long-term circulation characteristics, and metabolic discharge mechanisms that are superior to other biomaterials. These beneficial properties have resulted in the widespread use of polymer NPs as drug delivery systems and diagnostic contrast agents for medical applications. Despite their good biocompatibility, there are also disadvantages of polymer biomaterials in nano scale, especially under pathological conditions and the interactions of NPs with living cells are complex and still far from fully understood [38]. This article focuses on polymer NPs and explores their impact on the development of cardiovascular diseases such as AS and possible mechanisms of function.

Current research has shown that NPs < 100 nm in size are easily absorbed by tissues [39]. PLGA NPs prepared by dialysis were characterized by DLS, SEM and TEM and found to possess the expected size (nanoscale) and useful characteristics such as good dispersion, uniform size and spherical shape. Subsequently, we showed PLGA NPs efficiently bind serum proteins by SDS-PAGE and BCA kit. In addition to the standard physical criteria, medical biomaterials must also exhibit a high degree of compatibility with the circulatory system. Therefore, we evaluated blood compatibility of PLGA NPs from three aspects, hemolysis rate and coagulation function and platelet activation. The hemolysis rate of PLGA NPs was < 5%, in accordance with international standards. The physiological anticoagulant function is mainly achieved through the joint action of the coagulation system, platelets and the fibrinolysis system [40]. APTT mainly reflects the activity and function of endogenous coagulation factors, PT represents the exogenous coagulation system, TT is the time for conversion of fibrinogen to fibrin, Fbg is the content of fibrinogen, and GMP-140 indicates the activation of platelets. Through the detection of these five indicators, we found that the prepared PLGA NPs did not have a significant impact on coagulation and had excellent blood compatibility.

In 2017, Miller et al. studied the effects of gold NPs on cardiovascular disease, and discovered that red and purple particles accumulated in foam cells at sites of atherosclerotic plaque in ApoE^−/−^ mice treated with gold NPs [41]. Furthermore, gold NPs could be detected in surgical specimens of carotid artery disease from patients at risk of stroke. Based on previous research, in this paper we investigated the effects of PLGA NPs on AS by administrating PLGA NPs to ApoE^−/−^ mice by intravenous injection. We took ApoE^−/−^ mice as the animal model with cardiovascular risk factors, C57 mice as no any cardiovascular risk factors model. The ApoE^−/−^ mice were fed a HFD and injected with PLGA NPs for 4 or 12 weeks to investigate the short- or long-term effects of NPs on the development of AS. Our PLGA NPs long-term administration causes a significantly higher extension of plaque in AS animal model, ApoE^−/−^ mice with 12w HFD, but not in the C57 mice. And, it has been suggested that NPs could accumulate at sites of vascular disease, such as AS plaques [41]. Most NPs were be recruited by macrophages and accumulated in the inflammatory sites [38, 42]. According to the characteristics of AS plaque, the inflammatory cells would be increased at the plaque sites [43]. Here co-localization of PLGA NPs with the inflammatory marker CD68 and CD11b at the cross-sections of the aortic roots presented the accumulation of NPs at the plaque site compared with the negative C57 controls, no atherosclerotic plaques, no inflammatory cells. This result entailed that PLGA NPs had a close relationship with the inflammatory response then as shown in Fig. 4 PLGA NPs caused an increasing inflammatory factor release after 12w-injection.

In wild-type C57 mice with no plaque formation, immuno-histochemical staining detected only small amounts of the pro-inflammatory factors, TNF-α and IL-6 on the blood vessel walls and no expression of the anti-inflammatory factor IL-10. These observations indicated that in the absence of AS lesions (i.e., under normal physiological conditions) in mice, there was no significant inflammation and inflammatory factors were not activated and released. In the ApoE^−/−^ mice group that could spontaneously form AS plaques, strong positive expression of the pro-inflammatory cytokines TNF-α and IL-6 could be clearly observed at plaque sites. After 12 weeks of injection of PLGA NPs, TNF-α and IL-6 levels increased compared to the control groups. While IL-10 increased in all ApoE^−/−^ animals compared with C57 mice, but there were not significant differences between each ApoE^−/−^ groups and C57 groups. IL-10 is an indeed anti-inflammatory, changed with the progression and regression of inflammation [44–46]. This indicated that long term administration of NPs stimulate the release of inflammation factor but not company with the increase of anti-inflammatory factor. These results were consistent with recent studies regarding the relationships between inflammation and anti-inflammatory factors such as TNF-α/IL-10. Internal environmental stability is based on the dynamic balance between inflammatory and anti-inflammatory responses. When the inflammatory response dominates, tissues and cells will be damaged; whereas, a strong anti-inflammatory reaction will inhibit immune function [47]. An intricate balance between proinflammatory and anti-inflammatory cytokine signaling pathways that could leading to clinical events in the genesis of atherosclerotic lesions and in the probability of plaque rupture, such as myocardial infarction, stoke, and cardiovascular death.

During the injection process, NPs are rapidly coated with macromolecules forming a “protein crown or corona (PC)”, which alters the size, aggregation state, surface charge and interfacial properties of the nanomaterials to create a biological identity that is distinct from its original synthetic identity. PLGA NPs adsorbed a certain amount of proteins to form the PLGA + PC, with larger diameter and less stability, after incubation with mouse serum. NPs with protein coronas show completely different cell recognition or biological effects in vitro compared with in vivo [30].

Macrophages are the most important inflammatory cells in the process of AS lesion formation, are important components of lipid plaques, and serve as an important source of foam cells [48]. Therefore, we studied the effects of PLGA NPs on macrophages in vitro. According to the MTS assay results, the activity of Raw 264.7 cells decreased with increasing concentrations of PLGA NPs and PLGA + PC. The presence of the protein corona inhibited the phagocytosis of PLGA NPs by Raw 264.7 cells. Studies have shown that the role of the protein corona in biological systems can be divided into “opsonins” and “dysopsonins” [49]. Opsonins promote macrophage phagocytosis, while dysopsonins inhibit phagocytosis. The structure and composition of the corona depend on the synthetic identity of the nanomaterial, which includes the chemistry, topography and curvature of the nanomaterial. Polymer NPs possess various chemical compositions, free residues and morphologies (such as spheres, rods, vesicles, tubules and lamellae), which provide them with more diverse synthetic identities. After PLGA NPs enter the blood stream, the surface-adsorbed dysopsonins may be more abundant and more stable.

Hyperlipidemia is known to play an important role in the process of plaque formation. But our results indicated that PLGA NPs had no significant effect on lipid metabolism of both C57 and ApoE^−/−^ mice. While lipid transport, foam cell deposition also had key point function during the atherosclerotic development [50]. The physiological functions of proteins that comprise the protein corona include lipid transport, blood coagulation, complement activation, pathogen recognition and ion transport [51]. In the early stages of AS, ox-LDL acts as an inflammatory medium, promoting foam cell development and cholesterol-rich lipid core formation [52]. Cell ORO staining and CE/TC% suggested that PLGA NPs and PLGA + PC accelerated the conversion of Raw 264.7 cells to foam cells and that PLGA + PC had a stronger effect than PLGA NPs. Therefore, the protein corona absorbed on the surface of PLGA NPs may possess a stronger atherogenic potential. This phenomenon may explain many existing inconsistencies between in vitro toxicity screening and in vivo studies, and necessitate a re-evaluation of the toxicity of polymer NPs, even for polymer materials with good biocompatibility.

As an in vitro and animal in vivo study, it should be noted that the doses employed in the in vitro analysis were much higher than those used in vivo. The body is a whole circulatory system that metabolizes NPs and the internal and external environment is a completely different environment. We performed in vivo and in vitro experiments according to the concentrations used for nanotoxicity evaluation in the references [34, 36]. For this reason, we will pay more attention to this interesting information and try to find its mechanism in the future researches, we also should be much more circumspect when we make the conclusion from the animal experiment results, especially the gene KO mice.

## Conclusions

In this study, we observed that phagocytosis of polymeric NPs with good blood compatibility may cause a significantly higher extension of atherosclerotic plaques and have close relationship with many cardiovascular risk factors such as inflammation, and abnormal hemodynamics. The long-term administration of polymeric NPs may induce macrophage activation and transformation into foam cells, followed by an increase in inflammatory factors. The types of proteins and amount of protein corona absorbed on the surface of polymeric NPs may play a key role during this process. These in vivo animal results highlight the neglected hazard for polymeric NPs what we should not be ignored in future nanomaterial design and pay more attention to the process of using nano-medicines on cardiovascular diseases.

## Supplementary Information

**Additional file 1: Fig. S1.** SDS-PAGE Characterization of protein coronae formed on the surface of PLGA NPs exposed to serum. Lane 1: standard molecular marker, Lane 2: 10 μg PLGA + PC, Lane 3: 10 μg PLGA NPs, Lane 4: supernate after three cycles of washing and centrifugation for 10 μg PLGA + PC.** Fig. S2.** H&E staining of main organs after PLGA NPs injection for 4 and 12 weeks. There were no obvious changes between each group.

## Data Availability

All data generated or analyzed during this study are included in this published article and its additional information files.
