# Supplementary material for: Phagocytosis of polymeric nanoparticles aided activation of macrophages to increase atherosclerotic plaques in ApoE−/− mice
Source: J Nanobiotechnology. 2021 Apr 28;19:121. doi: 10.1186/s12951-021-00863-y (PMC8082811; doi:10.1186/s12951-021-00863-y)
Supplement: Supplementary file 1 — Additional file 1: Fig. S1. SDS-PAGE Characterization of protein coronae formed on the surface of PLGA NPs exposed to serum. Lane 1: standard molecular marker, Lane 2: 10 μg PLGA + PC, Lane 3: 10 μg PLGA NPs, Lane 4: supernate after three cycles of washing and centrifugation for 10 μg PLGA + PC. Fig. S2. H&E staining of main organs after PLGA NPs injection for 4 and 12 weeks. There were no obvious changes between each group. [file 12951_2021_863_MOESM1_ESM.docx]

Additional Information

**Phagocytosis of Polymeric Nanoparticles Aided Activation of**  **Macrophages to Induce Atherosclerotic Plaques in ApoE^-/-^ Mice**

Tieying Yin^1,^*, Yanhong Li^1^, Atik Rohmana Maftuhatul Fuad^1^, Yuzhen Ren^1^, Fangfang Hu^1^, Ruolin Du^1^, Yang Wang^1^, Guixue Wang^1^, Yazhou Wang^1,2,^*

^1^ Key Laboratory for Biorheological Science and Technology of Ministry of Education, State and Local Joint Engineering Laboratory for Vascular Implants, Bioengineering College of Chongqing University, Chongqing, 400044, China

^2^ School of medicine, Chongqing University, Chongqing, 400030, China


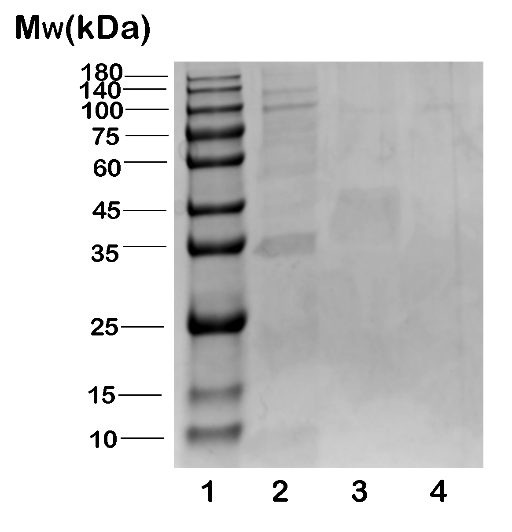


**Fig. S1.** SDS-PAGE Characterization of protein coronae formed on the surface of PLGA NPs exposed to serum. Lane 1: standard molecular marker, Lane 2: 10 μg PLGA+PC, Lane 3: 10 μg PLGA NPs, Lane 4: supernate after three cycles of washing and centrifugation for 10 μg PLGA+PC.


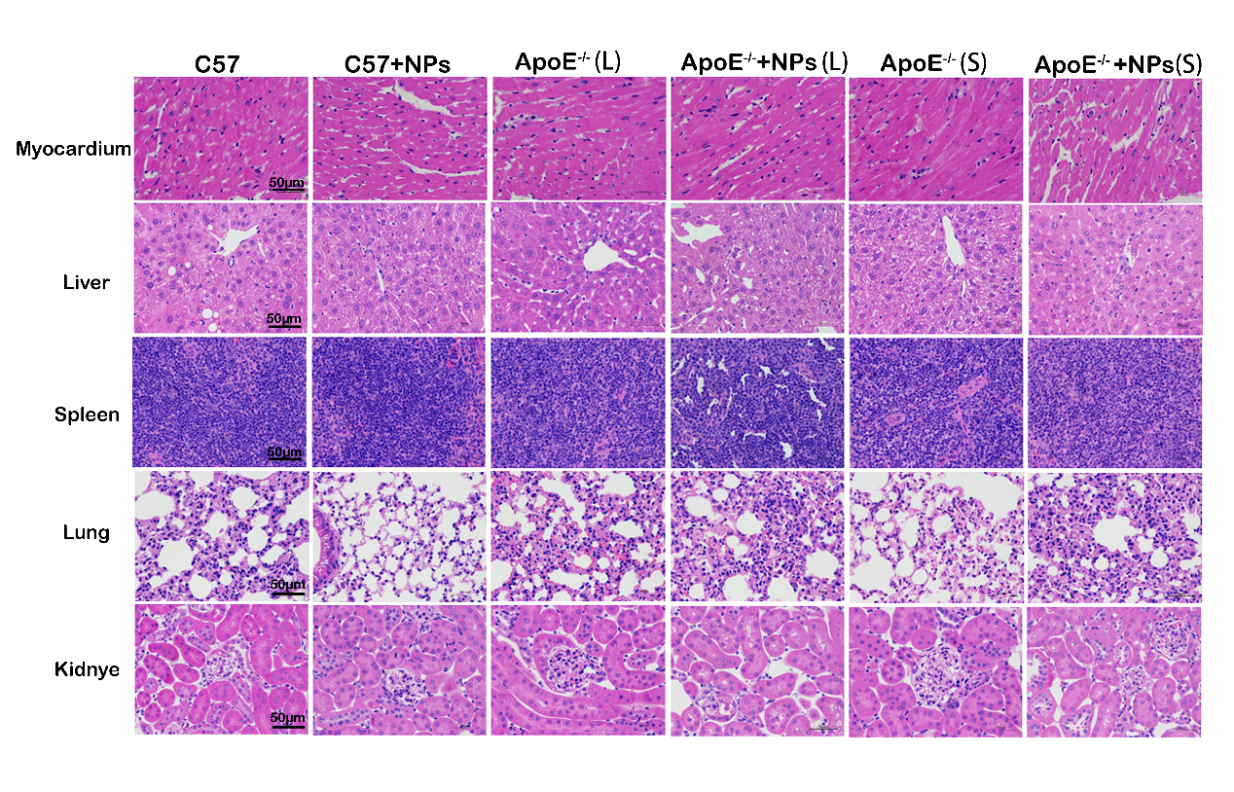


**Fig. S2.** H&E staining of main organs after PLGA NPs injection for 4 and 12 weeks. There were no obvious changes between each group.
